# Implementation of multilingual support of the European Multicenter Study about Spinal Cord Injury (EMSCI) ISNCSCI calculator

**DOI:** 10.1038/s41393-021-00672-y

**Published:** 2021-08-17

**Authors:** Christian Schuld, Steffen Franz, Joachim Schweidler, Jiri Kriz, Renata Hakova, Norbert Weidner, Rüdiger Rupp, Nan Liu

**Affiliations:** 1 Spinal Cord Injury Center, Heidelberg University Hospital, Heidelberg, Germany; 2 Spinal Cord Unit, Department of Rehabilitation and Sports Medicine, 2nd Faculty of Medicine, Charles University and University Hospital Motol, Prague, Czech Republic; 3 Department of Rehabilitation Medicine, Peking University Third Hospital, Beijing, China

**Keywords:** Translational research, Outcomes research

## Abstract

**Objectives:**

Since their introduction, electronic International Standards for Neurological Classification of Spinal Cord Injury (ISNCSCI) calculators have evolved to powerful tools providing error-free ISNCSCI classifications in education, research and clinical practice. For increased accessibility and dissemination, a multilingual support is mandatory. The aim of this work was to setup a general multilingual framework for the freely available ISNCSCI calculator (https://ais.emsci.org) of the European Multicenter Study about Spinal Cord Injury (EMSCI).

**Methods:**

The graphical user interface (GUI) and PDF export of the ISNCSCI worksheet were adapted for multilingual implementations. Their language-dependent content was identified. These two steps called internationalization have to be performed by a programmer in preparation of the translations of the English terms into the target language. This step following the internationalization is called localization and needs input by a bi-lingual clinical expert. Two EMSCI partners provided Standard Mandarin Chinese and Czech translations. Finally, the translations are made available in the application.

**Results:**

The GUI and PDF export of the ISNCSCI worksheet were internationalized. The default language of the calculator is set according to the user’s preferences with the additional possibility for manual language selection. The Chinese as well as a Czech translation were provided freely to the SCI community.

**Conclusions:**

The possibility of multilingual implementations independent from software developers opens the use of ISNCSCI computer algorithms as an efficient training tool on a larger scale.

## Introduction

The International Standards for Neurological Classification of Spinal Cord Injury (ISNCSCI) [1, 2] published by the American Spinal Injury Association (ASIA) and the International Spinal Cord Society (ISCoS) is based on a standardized clinical segmental examination of sensory and motor function. It provides classification procedures for determining the level and the severity of a spinal cord injury (SCI). The ISNCSCI examination consists of a bilateral manual muscle test of 5 myotomes on the arms (C5-T1) and 5 myotomes on the legs (L2-S1), a bilateral sensory test for light touch appreciation as well as pin prick discrimination in 28 dermatomes (C2-S4-5) and an anorectal examination of voluntary anal contraction of the external sphincter and deep anal pressure sensation. Intensive training has been strongly recommended to ensure a reliable examination [3, 4].

Based on this clinical examination, several classification variables have been defined describing the location of the SCI (neurological level of injury, motor and sensory levels), the severity of the SCI (complete versus incomplete, ASIA Impairment Scale) and the zones of partial preservation in cases with missing sensory or motor function in the most sacral segments. The classification steps are relatively complex, and the rules have undergone several revisions and updates [1]. Thus, appropriate training is recommended to ensure a correct determination of the ISNCSCI classification variables, particularly of motor levels and ASIA Impairment Scale [5–8]. Misclassification rates of manually determined motor levels range between 15% [6] and 38% [9] for clinicians not specifically trained in classification. Due to the complexity of the classification rules and the difficulties in manual classification, the need for electronic ISNCSCI calculators has been identified during the last decade [10]. Since then, ISNCSCI calculators have evolved into powerful tools. Two of them, the ISNCSCI computer algorithm [11] of the Rick Hansen Institute (https://www.isncscialgorithm.com/) and the ISNCSCI calculator of the European Multicenter Study about Spinal Cord Injury (EMSCI) [8] (https://ais.emsci.org) have been validated using large datasets and are freely available online. Both calculators contain a sophisticated inference logic for the consistent classification of datasets containing not-testable scores, which can be very challenging and time-consuming for clinicians to classify manually. In general, calculators assist clinicians and researchers in data management, in improving data quality and with up-to-date ISNCSCI classifications based on the most current ISNCSCI [8]. Considering the relevance of ISNCSCI as an internationally accepted and widely used assessment tool for clinical decision-making and research, there is a need of multilingual versions of the calculator beyond the established English version.

In computing, multilingual support is typically divided into the processes “internationalization” and “localization”, which are methods to adapt computer software to different languages, regional differences and technical requirements [12]. Internationalization (frequently abbreviated to the numeronym I18N) describes the process of designing a software application in a way that allows potential adaption to various languages and regions without changes to its core algorithmical parts [13]. Although this process needs to be performed only once, it is most often very time-consuming. This applies particularly to small software projects, when it was not considered by the software developers from the beginning, and might result in considerable restructuring of the software. Localization (numeronym L10N) is the process of adapting internationalized software for a specific region or language by adding locale-specific components and translating text [13]. This task is typically less time-consuming than the I18N task and translational skills are needed rather than developer skills.

Whenever a user interacts with the ISNCSCI calculator in a language other than English, I18N and L10N are involved. This includes the web-form to enter the ISNCSCI scores, the web-site in which the ISNCSCI web-form is embedded and the generated reports, e.g., the PDF worksheet based on the entered scores. Only if all these components of the calculator are internationalized and localized, the user can choose among different languages.

The aim of this work was (1) to internationalize the ISNCSCI calculator provided by EMSCI with fully freely available tools for support of multiple language versions, and (2) to localize the calculator with two languages. With the help of two EMSCI partners (Department of Rehabilitation Medicine, Peking University Third Hospital, Beijing, China; Spinal Cord Unit, Department of Rehabilitation and Sports Medicine, 2nd Faculty of Medicine, Charles University and University Hospital Motol, Prague, Czech Republic) the freely available EMSCI ISNCSCI calculator web application was translated to Standard Mandarin Chinese [14] and to Czech.

## Methods

From a technical perspective, the calculator consists of a front end containing the graphical user interface (GUI) and a back end for data storage and management and for performing all ISNCSCI classifications. Details on the ISNCSCI calculation algorithms used in the back end are published elsewhere [8]. For this work, multilingual support was implemented for the GUI and the ISNCSCI worksheet export, because all other parts were developed in English as the internationally accepted language of informatics. The ISNCSCI booklet updated 2015, revised 2011 [15] was used as basis for translations of GUI and worksheet (REV 04/15).

The “GNU gettext” toolkit [16] and the tinygettext toolkit [17] were used for I18N and L10N, respectively. The “GNU gettext”- toolkit provides the general internationalization framework, file formats and tools, whereas tinygettext is used for the actual localization of the application.

### Internationalization prerequisites

The first step in internationalization of software is the selection of a suitable character encoding used throughout the application. For some languages, e.g., English, a condensed character set like the ASCII (abbreviated from American Standard Code for Information Interchange) [18] encoding is sufficient. ASCII represents a fixed length (7-bit) encoding originally developed from telegraph code and consists of 95 printable characters (letters, digits, punctuation marks and a few miscellaneous symbols). However, this widely used encoding is not suitable for any internationalization project, because the number of available characters is optimized for Latin-based languages and especially for English.

Unicode is an industry standard for the consistent encoding, representation and handling of text expressed in most of the world’s writing systems. The latest (May 2019) Unicode standard 12.1 contains nearly 138.000 characters (https://unicode.org/versions/Unicode12.1.0/). UTF-8 (abbreviated from Universal Coded Character Set Transformation Format – 8-bit) [19] is the most frequently used Unicode character encoding and was chosen to replace the already implemented ASCII encoding because of its backward compatibility with ASCII, which minimizes changes in the source code.

### Internationalization of the ISNCSCI PDF worksheet

**Fig. 1:**
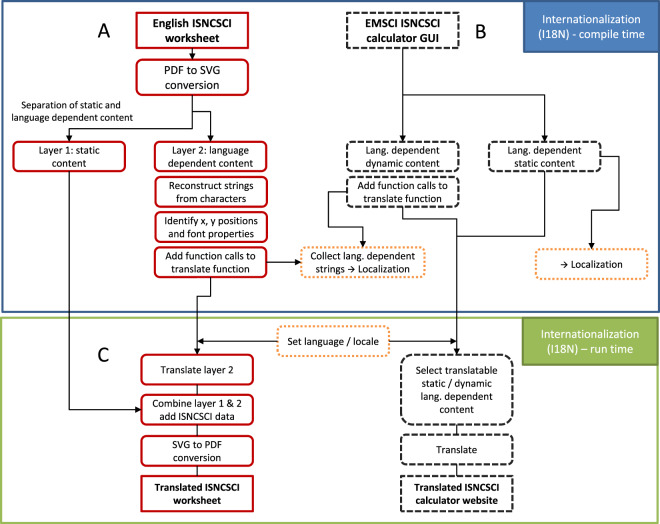
Overview of the workflow of the internationalization (I18N) of an ISNCSCI computer algorithm (here the EMSCI ISNCSCI calculator). Subfigure (**A**) represents the I18N of the ISNCSCI worksheet at compile time, whereas subfigure (**B**) depicts the I18N of the graphical user interface (GUI) of the EMSCI ISNCSCI calculator. The I18N steps at runtime are shown in subfigure (**C**) for the ISNCSCI worksheet and the EMSCI ISNCSCI calculator. The groups of the processing steps are color- and line-style coded: red (solid line) for the Internationalization of the ISNCSCI worksheet, black (dashed with short lines) for the EMSCI ISNCSCI calculator GUI and orange (dashed with dots) for steps commonly used in multiple tasks. Abbreviations: International Standards for Neurological Classification of Spinal Cord Injury (ISNCSCI), European Multicenter Study about Spinal Cord Injury (EMSCI).

As the electronic ISNCSCI worksheet (Fig. 1A) is only available as Portable Document File (PDF) [20], the first processing step for internationalizing was the conversion from the PDF format into a more editable file format. In general, PDF is a file format independent of the application software, hardware and operating system mainly intended for storage, viewing and printing, but not for editing [20]. To generate an editable worksheet representation we chose to convert the PDF format into the Scalable Vector Graphics (SVG) file format. SVG is a text-based open vector image format for two-dimensional graphics with support for interactivity and animation [21]. INKSCAPE (free and open source software licensed under the GNU General public license (GPL), https://www.inkscape.org) was used to convert the PDF-version of the worksheet into SVG. The SVG worksheet representation was divided into two layers: The first layer contains all static, language-independent content. This includes mainly the dermatome map, the grids and all boxes for the examination scores. In the second layer, all translatable content is bundled. In general, typical word processing tasks like text selection or copy & paste might not be possible in PDF-documents, because words might not be represented as grouped characters (called “string” in computing), but rather as a bunch of single, not linked characters. In particular, the original ISNCSCI worksheet PDF contained only unlinked characters. Accordingly, the most labor-intensive step was to build strings from the single characters. For every string, the exact horizontal and vertical position on the worksheet as well as the font properties (type, size, and weight) were determined to assure the same worksheet design in the SVG document as in the original PDF document. For quality control and to identify layout errors, the new SVG document was overlaid to the original PDF document. In an iterative process, erroneous items and differences in the SVG document were adjusted by the authors CS and JS until the senior author RR was not able to detect any differences by visual inspection.

In a last step, every string representing an English word in the SVG document was marked as translatable to allow for replacement during the localization process.

### Internationalization of the EMSCI ISNCSCI calculator GUI

The EMSCI ISNCSCI calculator GUI (Fig. 1B) can be divided into more or less static content, which is served from hypertext markup language (HTML) files (disclaimer, welcome, history, team and manual text pages), and dynamically created content (main calculator user interface and examples page). These two groups of content are mapped into two processes in I18N (Fig. 1B): The HTML files with static content are translated en bloc. An international language code suffix is added to the filename containing the translated text, e.g., the Chinese translated welcome page is saved as “Welcome_zh.html” where “zh” is the international (ISO 639-1) language code for Sino-Tibetan/Chinese languages. The international language code for Czech is “cs”. Accordingly, the Czech welcome page is named “Welcome_cs.html”.

For the dynamically created content like in the ISNCSCI worksheet, all translatable content is marked for the subsequent localization process.

### Internationalization at run time of the EMSCI ISNCSCI calculator

**Fig. 2:**
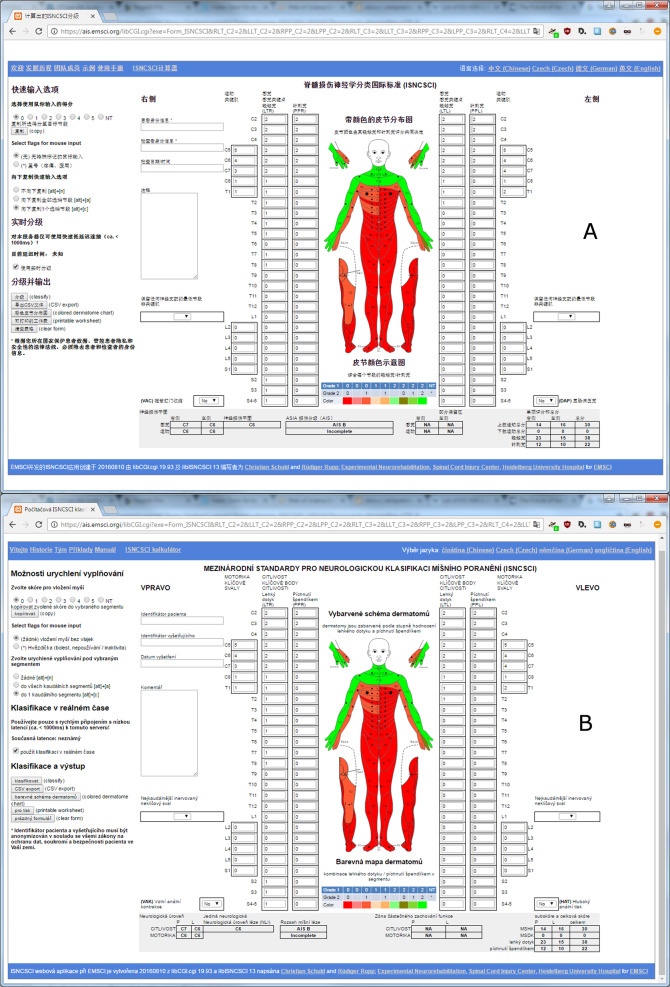
Graphical user interface of the EMSCI ISNCSCI calculator. Subfigure (**A**) depicts the Chinese localization and subfigure (**B**) the Czech localization. The ISNCSCI classification depicted on this figure was done according to ISNCSCI revised 2011, updated 2015 [15]. The zones of partial preservation would be T1 (right motor ZPP), T1 (left motor ZPP), not applicable (right sensory ZPP) and T2 (left sensory ZPP) if the current 2019 ISNCSCI [2] would have been used. Abbreviations: European Multicenter Study about Spinal Cord Injury (EMSCI), International Standards for Neurological Classification of Spinal Cord Injury (ISNCSCI).

When a user visits the calculator website for the first time, the default language is set according to the user’s web-browser settings. Technically, web-browsers send a configurable weighted list of preferred languages upon requesting a website [22], e.g., in the list “cs;q=0.8,en-US;q=0.6,en;q=0.4.” Czech (“cs”) is preferred over American English (“en-US”) and over English in general (“en”). The calculator selects the supported language having the highest weight. If none of the browser’s announced languages is supported, English is chosen as default language. The selected language is stored as a cookie in the web browser and used for subsequent visits. Additionally, links to manual language selection are provided in the user interface (Fig. 2). A click on one of these links sets the language and immediately reloads the site to apply the language change.

The PDF of the ISNCSCI worksheet is produced during runtime of the web application (Fig. 1C) by converting the translated SVG document to a PDF document using a non-interactive command line call to INKSCAPE on the hosting server. The translated SVG document consists of three layers: The first layer contains all static, language-independent elements (grids, dermatome charts, etc.). The second layer contains all translatable items and is dynamically created for the selected language. The third layer is also dynamically created and contains the examination scores as well as the calculated ISNCSCI classification results, which however are not translated at this time (e.g., “AIS B” stays “AIS B” in every language).

### Internationalization - message catalog

The English words and phrases marked as translatable in the language-dependent parts of the GUI and the ISNCSCI PDF (Table 1) were automatically collected from the source code by the “xgettext” tool. This message catalog is a major outcome of the internationalization process and provides the basis for the subsequent localization processes.

**Table 1:** Table containing a subset of English terms and their translations (Chinese and Czech translations) used for dynamical creation of the ISNCSCI calculator GUI and PDF worksheet.

Portable Object Template ISNCSCI.pot	Chinese translation zh_CN.po	Czech translation cs_CZ.po	To be continued for other languages …
Elbow flexors	肘屈肌	Flexory lokte	
Wrist extensors	腕伸肌	Extenzory zápěstí	
Elbow extensors	肘伸肌	Extenzory lokte	
Finger flexors	指屈肌	Flexory prstů	
Finger abductors	指外展肌	Abduktory prstů	
little fingers	小指	Malík	
COMPLETE OR INCOMPLETE?	完全性或不完全性?	KOMPLETNÍ NEBO NEKOMPLETNÍ?	
KEY MUSCLES	关键肌	KLÍČOVÉ SVALY	
MOTOR	运动	MOTORIKA	
SENSORY	感觉	CITLIVOST	
KEY SENSORY POINTS	感觉关键点	KLÍČOVÉ BODY CITLIVOSTI?	
(VAC) Voluntary anal contraction	(VAC) 随意肛门收缩	(VAK) Volní anální kontrakce	
Non-key Muscle?	非关键肌?	Neklíčový sval?	
ASIA Impairment Scale	ASIA 损伤分级 (AIS)	ASIA Rozsah míšní léze	

### Localization of the EMSCI ISNCSCI calculator

For localization, this catalog was translated using Poedit (Copyright 2016 Václav Slavík, https://poedit.net), which is a free cross-platform graphical gettext translation editor. For each supported language, a so-called portable object (*.po) file is derived from message catalog’s portable object template (*.pot) containing all above mentioned English words and phrases. The editable files are named using the international language code as prefix, e.g., zh_CN.po for the Chinese portable object file.

For the translation of the static HTML-files, any text editor with Unicode (UTF-8) support can be used.

### Translation

The Spinal Cord Unit at the Department of Rehabilitation and Sports Medicine of the 2nd Medical Faculty and University Hospital in Motol was responsible for the translation into Czech language. The process of the translation followed recommendations from Biering-Sørensen et al. [23] and a back translation into the original Czech language was performed by an independent person. The translated 2013 ISNCSCI revision was published in peer-reviewed Czech journal [24]. All subsequent changes, as well as the translation of the document for this manuscript, have been regularly re-checked and adapted using the same translation procedure. The Chinese translation was first conducted for the 2015 update of the ISNCSCI booklet [25] including the worksheet. This translation was reviewed by a native speaker from an independent institution in the field of SCI medicine. All additional translations for the GUI of this project have been conducted by three experienced experts, but no formal back translation or independent review was conducted. Author NL managed the Chinese translation, authors JK and RH the Czech translation. None of them had a professional software development education.

Several reviewing rounds between the programmers (CS and JS) and the translators were needed, until all spelling issues, grammar issues and language issues (phrases like “sensory level”, “motor level” versus three single words “sensory”, “motor” and “level”) have been resolved. In each round either the programmers had to solve I18N problems in the application and/or the translators had to solve L10N problems. Eventually, the translators have to approve the correctness of the translations in the application which concluded the reviews.

## Results

The GUI of the EMSCI ISNCSCI calculator and the PDF export of the ISNCSCI worksheet were successfully internationalized. As a proof-of-concept of the multi-language support, two non-English versions of EMSCI ISNCSCI calculator were implemented. First, a Chinese version was launched at the annual meeting of the American Spinal Injury Association (ASIA) 2016 in Philadelphia, USA [26] and is freely available online (https://ais.emsci.org). The collaboration for the Czech translation was established at the annual meeting of the International Spinal Cord Society (ISCoS) 2016 in Vienna, Austria [27]. This translation will be incorporated into the next major update of the EMSCI ISNCSCI calculator.

**Fig. 3:**
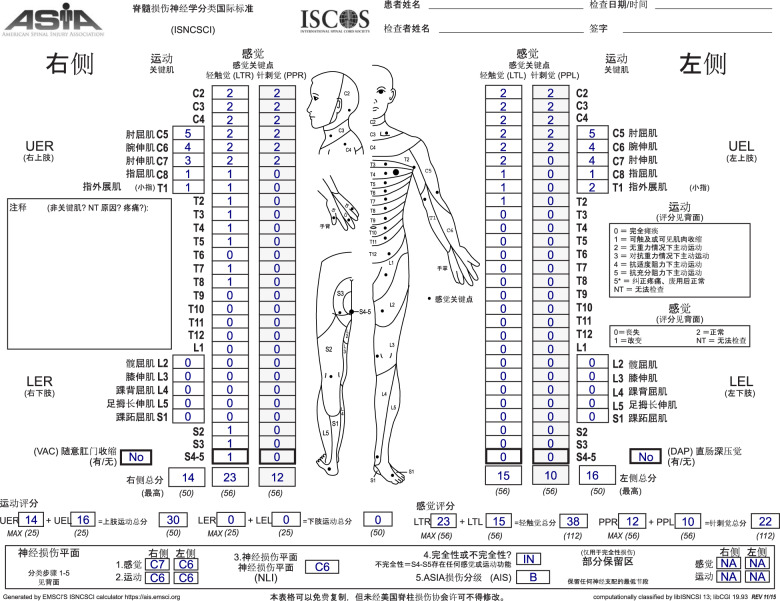
Dynamically generated Chinese version of the ISNCSCI worksheet. The worksheet is generated as a pdf document from an example case entered in the EMSCI ISNCSCI calculator. The ISNCSCI classification depicted on this figure was done according to ISNCSCI revised 2011, updated 2015 [15]. The zones of partial preservation would be T1 (right motor ZPP), T1 (left motor ZPP), not applicable (right sensory ZPP) and T2 (left sensory ZPP) if the current 2019 ISNCSCI [2] would have been used. Abbreviations: European Multicenter Study about Spinal Cord Injury (EMSCI), International Standards for Neurological Classification of Spinal Cord Injury (ISNCSCI).

**Fig. 4:**
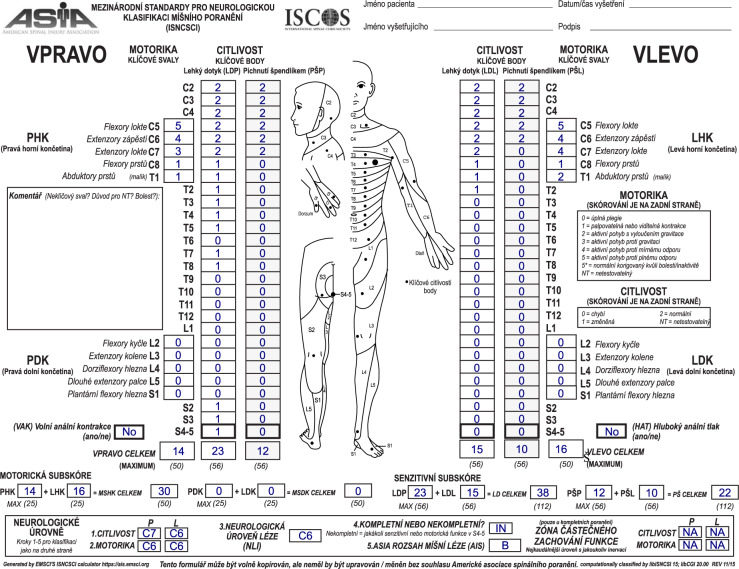
Dynamically generated Czech version of the ISNCSCI worksheet. The worksheet was generated a pdf document from an example case entered in the EMSCI ISNCSCI calculator. The ISNCSCI classification depicted on this figure was done according to ISNCSCI revised 2011, updated 2015 [15]. The zones of partial preservation would be T1 (right motor ZPP), T1 (left motor ZPP), not applicable (right sensory ZPP) and T2 (left sensory ZPP) if the current 2019 ISNCSCI [2] would have been used. Abbreviations: European Multicenter Study about Spinal Cord Injury (EMSCI), International Standards for Neurological Classification of Spinal Cord Injury (ISNCSCI).

The Chinese translation covers the complete website including the GUI as well as the PDF export of the front side of the ISNCSCI worksheet. At the current stage, the Czech translation covers only the ISNCSCI calculator GUI and the PDF export. Screenshots of the Chinese and Czech calculator GUIs are shown in Fig. 2A, B. The corresponding PDF exports of ISNCSCI worksheet are depicted in Figs. 3 and 4, respectively.

The I18N message catalog consists of 182 English words and phrases, which need to be translated once into the respective language (Table 1). The time needed once to translate these 182 terms is estimated to be less than 1 h. This step was performed so quickly, because the Chinese as well as Czech ISNCSCI relevant translations were already available from other projects [24]. There is already a Chinese translation of the ISNCSCI worksheet officially endorsed by ASIA and ISCoS available [25].

After the translation of the message catalog, there were only small adaptions necessary to the SVG worksheet to avoid overlapping of neighboring elements. The Chinese I18N needed some special processing, e.g., smaller and only normal weighted fonts. This is in contrast to the original English version, which contains a mix of oblique, normal, italic and bold weighted fonts. For the Czech I18N, only a smaller font size for the total light touch, pin prick and motor scores was necessary together with slight modifications of the position of some headings.

## Discussion

With this work, the technical basis for multilingual versions of the electronic ISNCSCI calculator has been established. As a proof-of-concept, two versions in Chinese and Czech language were implemented. The concepts of internationalization and localization allow for adding a new language to the calculator in the most effective way, because the translators can operate mostly independent of software developers. All software tools needed for internationalization of the EMSCI ISNCSCI calculator are free and open source ensuring that the calculator can be offered to the SCI community for free as well as on a long-term basis.

A Chinese localization fosters the use of ISNCSCI calculators for education of the Chinese speaking clinicians among the approximately 900 million first-language Chinese speaking people. A website available in the users’ native language does not only increase the perceived usability [28], but also enables users having problems correctly understanding English terms and phrases or even language anxiety [29] to make full use of this website. Although English is the internationally accepted world language in particular in research [30], there is still a need for translations of assessments into local languages even in countries where English represents the second language [14].

The main goal of the EMSCI ISNCSCI calculator is to support clinicians and researchers in the correct classification of difficult ISNCSCI cases. It has to be clearly stated that it is not intended as a replacement of the manual classification. It is essential that clinicians maintain their classification skills to identify those elements of the examination that are most crucial for a correct classification of an individual with SCI [7].

As an additional benefit of the internationalization of the EMSCI ISNCSCI calculator, high quality, electronic ISNCSCI worksheets in SVG format in the respected language can be created very easily. These translated ISNCSCI worksheets represent an important part in the translation process of the ISNCSCI documents such as the ISNCSCI booklet [15].

The I18N has a technical limitation regarding text directionalities, i.e., text is written right-to-left (or dextrosinistral), left-to-right (or sinistrodextral) or boustrophedon (changes of text directionality after each row). At this time, only sinistrodextral languages written from left-to-right are supported. The translators cannot upload their translations directly into calculator, which needs to done manually by a system administrator.

### Future work

This work is based on the ISNCSCI version revised 2011 and updated in 2015 [15]. For the next release of the calculator, it is planned upgrading to the 2019 current ISNCSCI revision [1]. However, the upgrade will not only include work on the internationalization part but also major changes to the algorithmical/computational framework, e.g., regarding the non-SCI taxonomy [31] and the revised Zones of Partial Preservation. The EMSCI ISNCSCI calculator represents a major component in a wider context to improve the quality of ISNCSCI under the umbrella of the EMSCI network [7–10, 32–35]. The calculator has been actively maintained and developed since 2003. The long history as well as the membership of two authors (CS and RR) in the ASIA International Standards Committee shows that there is an intention for long-term continuation of this work.

With the implementation of the multilingual support, it is planned to introduce more languages in the future into the EMSCI ISNCSCI calculator so that more people can get access to educational tools supporting the correct classification of ISNCSCI examinations.

We believe that not only the front side of the ISNCSCI worksheet should be part of the translation process, but also the back side, containing a very condensed set of ISNCSCI examination and classification rules. The implementation of this feature is planned for a future release.

## Conclusion

The EMSCI ISNCSCI calculator has been internationalized and successfully localized to Chinese and Czech versions. As part of the internationalization process, a framework for implementation of multilingual versions based on static content and message catalogs used for dynamic website content has been set up, which does not depend on the support of software developers. This contributes to an increased dissemination of electronic ISNCSCI computer algorithms. With the introduced version, the algorithms are easier to access for better training of examiners and for correct classification of ISNCSCI datasets.
